# A Cleavable N-Terminal Signal Peptide Promotes Widespread Olfactory Receptor Surface Expression in HEK293T Cells

**DOI:** 10.1371/journal.pone.0068758

**Published:** 2013-07-01

**Authors:** Blythe D. Shepard, Niranjana Natarajan, Ryan J. Protzko, Omar W. Acres, Jennifer L. Pluznick

**Affiliations:** Department of Physiology, Johns Hopkins University School of Medicine, Baltimore MD, USA; German Institute for Human Nutrition, Germany

## Abstract

Olfactory receptors (ORs) are G protein-coupled receptors that detect odorants in the olfactory epithelium, and comprise the largest gene family in the genome. Identification of OR ligands typically requires OR surface expression in heterologous cells; however, ORs rarely traffic to the cell surface when exogenously expressed. Therefore, most ORs are orphan receptors with no known ligands. To date, studies have utilized non-cleavable rhodopsin (Rho) tags and/or chaperones (i.e. Receptor Transporting Protein, RTP1S, Ric8b and G_αolf_) to improve surface expression. However, even with these tools, many ORs still fail to reach the cell surface. We used a test set of fifteen ORs to examine the effect of a cleavable leucine-rich signal peptide sequence (Lucy tag) on OR surface expression in HEK293T cells. We report here that the addition of the Lucy tag to the N-terminus increases the number of ORs reaching the cell surface to 7 of the 15 ORs (as compared to 3/15 without Rho or Lucy tags). Moreover, when ORs tagged with both Lucy and Rho were co-expressed with previously reported chaperones (RTP1S, Ric8b and G_αolf_), we observed surface expression for all 15 receptors examined. In fact, two-thirds of Lucy-tagged ORs are able to reach the cell surface synergistically with chaperones even when the Rho tag is removed (10/15 ORs), allowing for the potential assessment of OR function with only an 8-amino acid Flag tag on the mature protein. As expected for a signal peptide, the Lucy tag was cleaved from the mature protein and did not alter OR-ligand binding and signaling. Our studies demonstrate that widespread surface expression of ORs can be achieved in HEK293T cells, providing promise for future large-scale deorphanization studies.

## Introduction

Olfactory receptors (ORs) are seven transmembrane domain G protein-coupled receptors (GPCRs) that govern the sense of smell in the olfactory epithelium, and comprise the largest gene family in the genome (~1000 OR genes in mice [1] and ~300 [2] in humans). Although this family was first identified over 20 years ago [3], the majority of ORs remain orphan receptors, with no known ligand. This is due, in large part, to the fact that OR deorphanization is typically attempted using *in vitro* ligand screening assays in heterologous cell systems which require surface expression of the OR as a prerequisite for the assay (i.e. HEK293T cells or *Xenopus* oocytes) [4–7]. Unfortunately, many ORs do not traffic to the cell surface in heterologous cell systems; rather, they are retained in the ER and degraded [8–10], making ligand assignment impossible. To combat this problem, studies have utilized the co-expression of various accessory proteins and/or the addition of N-terminal tags [11–14]. For example, the addition of the first 20 amino acids of rhodopsin onto the N-terminus of ORs (Rho tag) enhances OR surface expression for a number of ORs [15]. Similarly, receptor transporting protein (RTP), originally identified as a potential chaperone for ORs [16,17], also enhances expression of multiple ORs. A recent study showed that the best surface expression was achieved [18] by co-expressing the short form of RTP (RTP1S) [19], Ric8b (a putative GEF) [20] and G_αolf_ (the G protein that couples to ORs in the olfactory epithelium) [21] with Rho-tagged ORs. While these tools have been beneficial to the field [5,15,16,18,20,22–24] and are the most reliable enhancers of OR surface expression available to date, their effects are not universal. Despite these developments, many ORs are still unable to reach the cell surface when heterologously expressed, and thus remain as orphan receptors.

As membrane proteins, ORs enter the biosynthetic pathway upon translocation into the endoplasmic reticulum (ER). Typically, this is accomplished co-translationally where a signal peptide serves to mediate ER translocation through the heterotrimeric Sec61 complex that forms a channel in the ER membrane [25]. While most GPCRs use one of their transmembrane domains (TMD) as a signal anchor sequence, a small subset of GPCRs and other TMD proteins (and all secretory proteins) have cleavable signal peptides which are found at the extreme N-terminus of the immature protein [26,27]. As their name implies, these cleavable signal peptides are not incorporated into the mature protein; rather they are cleaved off in the ER membrane upon translocation. While cleavable signal peptides do not have a conserved sequence, they do share characteristic features including a hydrophobic region flanked by polar amino acids [25,26].

Recently, the single-spanning membrane protein, Leucine Rich Repeat Containing 32 (LRRC32) was found to possess a leucine-rich 17-amino acid cleavable signal peptide (MRPQILLLLALLTLGLA) which is required for proper ER translocation and surface expression in both T regulatory cells (where it is natively expressed) as well as in HEK293T cells [28]. Because the addition of other cleavable signal peptides has been shown to enhance surface expression for some GPCRs in cell culture [29,30], we hypothesized that the addition the LRRC32 signal peptide may promote surface expression of ORs. Importantly, as signal peptides are cleaved off in the ER, the addition of such a tag would not affect the mature protein, preventing any potential alteration or interference with ligand binding. To assay whether the addition of a cleavable signal peptide could aid in OR surface expression, we added the 17 amino acid signal peptide from LRRC32 (which we named “Lucy,” for its leucine repeats) to the N-terminus of 15 diverse ORs (murine ORs from both Class I and Class II, representing 11 different subfamilies, as well as 2 human ORs) and assayed for surface expression. We also combined our Lucy tag with both the Rho tag and the best practice in OR trafficking (co-expression with accessory protiens RTP1S, Ric8b and G_αolf_ [18]) in order to assess the universal effects of this tag. Here we report that the Lucy tag, in combination with the Rho tag and the accessory proteins, promotes surface expression of all ORs tested, raising the possibility for wide-spread deorphanization.

## Materials and Methods

### Reagents and Antibodies

Polyclonal (F7425) and M2 monoclonal (F1804) Flag antibodies and M2 Flag beads were purchased from Sigma (St. Louis, MO). The monoclonal HA antibody (3F10) was purchased from Roche (Indianapolis, IN) and the β-actin antibody was purchased from Abcam (Cambridge, MA). Alexa-conjugated fluorescent secondary antibodies were purchased from Invitrogen (Carlsbad, CA). HRP-conjugated secondary antibodies were purchased from Jackson ImmunoResearch Labs (West Grove, PA). The Dual-Luciferase Reporter Assay kit was purchased from Promega (Madison, WI). The odorants used in this study, isovaleric acid and eugenol, were also purchased from Sigma.

### OR Constructs and Cloning

The mOR-EG full-length construct, containing N-terminal Flag/Rho tags, was a kind gift from Kazushige Touhara (Univ. of Toyko) [22]. To clone the full-length sequences of the other ORs tested into the “Rho-OR” vector, the sequence encoding mOR-EG was excised from its parent vector (pME18S) and PCR products containing the full-length sequence of other ORs of interest were ligated into the corresponding sites in this vector. ORs were ligated in frame with an upstream start site, such that they incorporated sequences encoding N-terminal Flag and Rho tags. Full-length human OR51E2 and OR52B2 were amplified by PCR from human DNA (using primers which added appropriate restriction sites), taking advantage of the fact that ORs do not contain introns. The constructs containing murine Olfr78 (MOR18-2), Olfr90 (MOR256-21), Olfr1392 (MOR256-25), Olfr1393 (MOR256-24) and mOR-EG have been previously described [31]. The other full-length ORs were amplified using primers which added appropriate restriction sites from either mouse genomic DNA or from kidney RNA after performing RT (Olfr145 (MOR161-6, K21), Olfr99 (MOR156-1), Olfr394 (MOR135-8), Olfr545 (MOR42-1, S50), Olfr691 (MOR31-6), Olfr693 (MOR283-8), Olfr805 (MOR110-4), and Olfr985 (MOR171-4)). All constructs used in this study contain a Flag tag for detection purposes but will be referenced based on their other N-terminal tags for simplicity. All constructs were sequenced to confirm identity.

The Lucy tag (atgagaccccagatcctgctgctcctggccctgctgaccctaggcctggct) was added to the original (Rho-OR) vector for Olfr691 using overlap-extension PCR [32] to obtain a Lucy-Rho-OR. Subsequently, Olfr691 was excised from the Lucy-Rho vector, and the other ORs were excised from the original parent vector (Rho-OR) using the same restriction sites. The other ORs were then subcloned into the Lucy-Rho vector by ligation.

The Rho tag was deleted from both the Rho-Olfr78 and Lucy-Rho-Olfr78 constructs to obtain the OR or Lucy- OR constructs using PCR-mediated deletion [33]. Subsequently, Olfr78 was excised from the vectors, and the other ORs were excised from the original parent vector (Rho-OR) using the same restriction sites and then subcloned into the OR and Lucy- OR vectors by ligation.

To assay Lucy cleavage, an HA tag was added to the extreme N-terminus of the Lucy-Rho construct for Olfr691 using overlap-extension PCR [32], yielding an HA-Lucy-Flag-Rho-Olfr691 construct. As a control, an HA tag was also added to the extreme N-terminus of the Rho construct for Olfr691, yielding an HA-Flag-Rho-Olfr691 construct.

### Immunofluorescence

**Figure 1:**
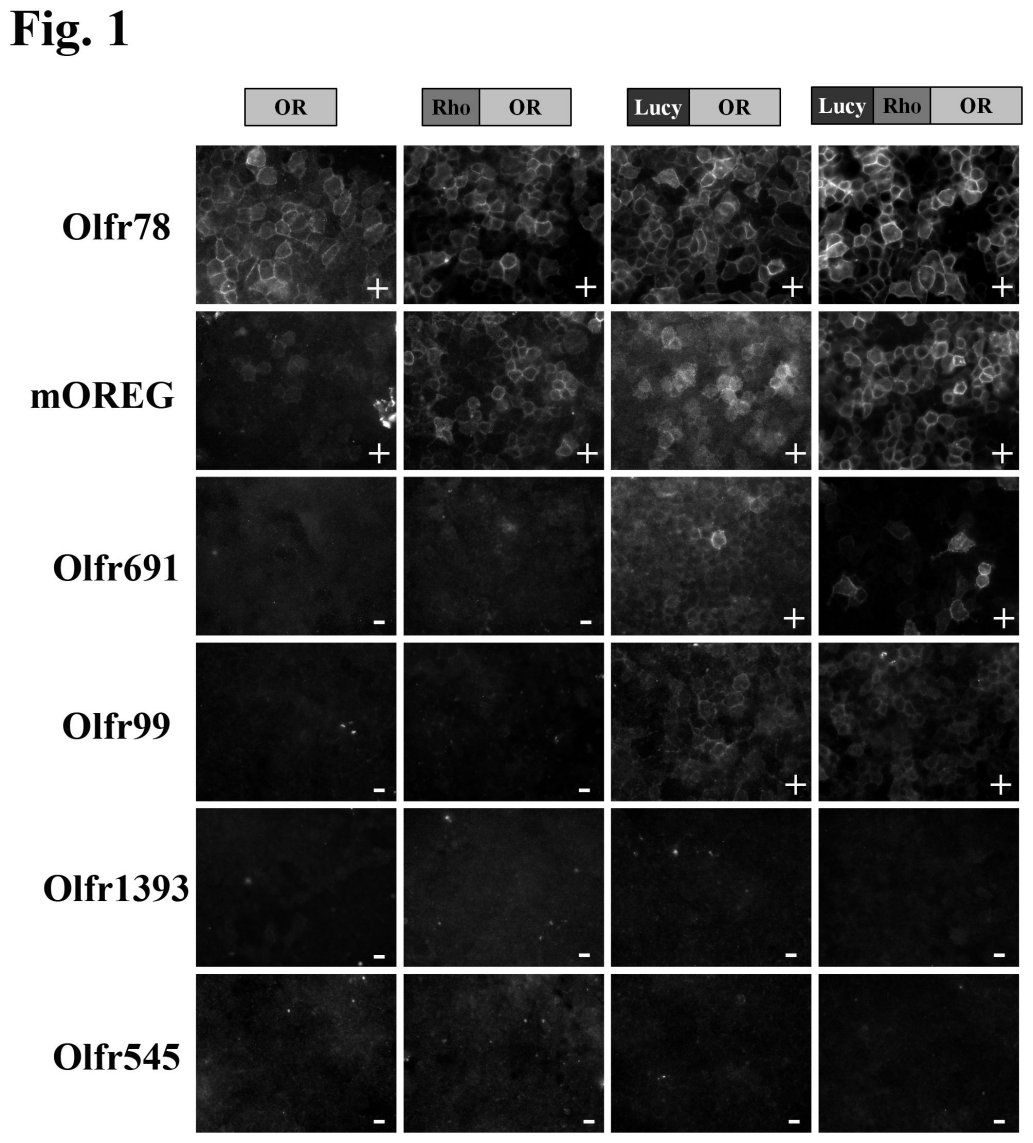
The Lucy tag aids in surface expression of some ORs in the absence of accessory proteins or the Rho tag. ORs were cloned and expressed in HEK293T cells without Rho or Lucy tags (OR), with a Rho tag (Rho-OR), with the Lucy tag (Lucy-OR) or with both the Lucy and Rho tags (Lucy-Rho-OR). The cells were then surface labeled with a Flag antibody to detect membrane-associated OR. Images were taken for each OR at equal exposure for all conditions. To assess OR surface expression, the entirety of each coverslip was systematically scanned and scored based on detectable surface immunofluorescence. A ‘+’ was scored for those ORs whose surface expression was detectable in >90% of all fields of view while ORs received an ‘*’ if surface expression was found in <50% of all fields of view. A complete lack of detectable surface expression was scored as a ‘-‘. Results for 6 representative ORs are shown in Figure 1 and the results for all ORs tested are summarized in Table 1.

**Figure 2:**
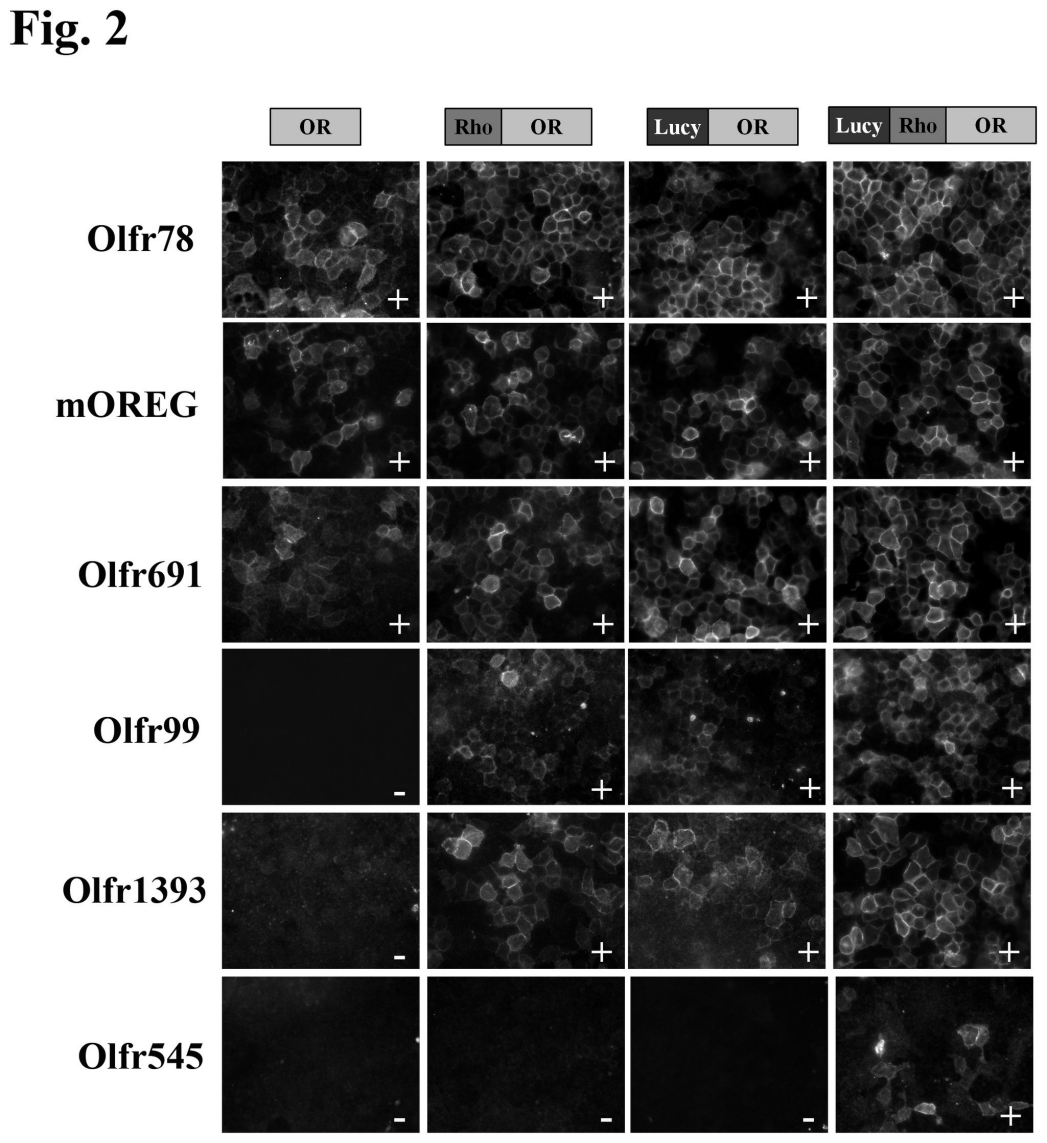
The Lucy tag works synergistically with accessory proteins and tags to promote surface expression of ORs.

HEK293T cells were seeded onto 18-mm coverslips coated with poly-L-lysine and transfected with OR constructs with or without accessory proteins (Lipofectamine 2000, Invitrogen). Flag-tagged OR trafficking was assayed using surface immunocytochemistry, as previously described [5]. Briefly, live, non-permeabilized cells at 4^o^C were exposed to a rabbit polyclonal anti-Flag antibody in PBS with 0.1% BSA. Subsequently, cells were washed, fixed with 4% paraformaldehyde, permeabilized (0.3% Triton X-100) and then exposed to a mouse monoclonal (M2) anti-Flag antibody. As the external Flag epitope (surface Flag) epitope is ‘blocked’ after binding to the polyclonal Flag antibody, the monoclonal Flag antibody detects only the internal population of ORs. Control experiments where the cells were surface labeled with the polyclonal Flag antibody followed by another surface label with the monoclonal Flag antibody confirmed that 100% of the external Flag epitopes were bound by the polyclonal antibody, as no subsequent labeling was seen with the second surface label. Fluorescent secondary antibodies reported the localization of the polyclonal and monoclonal Flag-tags. Cells were visualized for epifluorescence using a Zeiss Axiophot microscope (Thornwood, NY). Images were taken with a CoolSnap Digita Camera (Photometrics, Tuscon, AZ) and IP Labs software (Biovision, Exton, PA). For some experiments, a total of 0.8 µg of accessory plasmids (pcDNA3-RTP1S (modified from RTP1L, kind gift from S. Firestein, Columbia Univ.), pCMV Sport6-Ric8b (purchased from Open Bioystems), and pcDNA3.1-G_αolf_ (subcloned from a pGEMHE2 G_αolf_ construct, kind gift from S. Firestein, Columbia Univ.)) or 0.8 µg of an empty pcDNA4.1 vector (OR alone) were co-transfected along with 0.8 µg of the OR. In Figures 1 and 2, the immunofluorescent images shown in each row (OR, Rho-OR, Lucy- OR and Lucy-Rho- OR with and without accessory proteins) were transfected and processed simultaneously and image exposures remained constant for each OR. Images represent representative fields of view from at least 4 independent experiments. To assess OR surface expression, the entirety of each coverslip was systematically scanned and scored based on detectable surface immunofluorescence. A ‘+’ was scored for those ORs whose surface expression was detectable in >90% of all fields of view while ORs received an ‘*’ if surface expression was found in <50% of all fields of view. A complete lack of detectable surface expression was scored as a ‘-‘. To quantitate cell surface expression using ImageJ (Figure S2), the background was subtracted from the surface labeled Olfr691 images and the mean fluorescence intensity was measured. The mean intensity was normalized to the mean intensity of the corresponding binary nuclear image, to control for cell number.

ORs were cloned and co-expressed in HEK293T cells without Rho or Lucy tags (OR), with a Rho tag (Rho-OR), with the Lucy tag (Lucy-OR) or with both the Lucy and Rho tags (Lucy-Rho-OR) along with the chaperone proteins, RTP1S, Ric8b and G_αolf_. The cells were then surface labeled with a Flag antibody to detect membrane-associated OR. Images were taken for each OR at equal exposure for all conditions. To assess OR surface expression, the entirety of each coverslip was systematically scanned and scored based on detectable surface immunofluorescence. A ‘+’ was scored for those ORs whose surface expression was detectable in >90% of all fields of view while ORs received an ‘*’ if surface expression was found in <50% of all fields of view. A complete lack of detectable surface expression was scored as a ‘-‘. Results for 6 representative ORs are shown in Figure 2 and the results for all ORs tested are summarized in Table 1.

### Enzyme-Linked Immunosorbent Assay (ELISA)

ELISA measurements in HEK 293T cells transfected with various constructs were performed as previously described [5,34]. Wells for ELISA were assayed in quadruplicate. Briefly, transfected cells seeded in a 96 well plate were fixed and permeabilized. OR-expressing cells were probed with the polyclonal Flag antibody and detected with anti-rabbit HRP-conjugated secondary antibody. HRP levels were detected with 1-Step Ultra TMB (3,3’,5,5’-tetramethylbenzidine) (Thermo Scientific, Rockville, IL).

### Immunoprecipitation and Western Blotting

HEK293T cells in 35-mm dishes were transfected for 24 h with either the Rho- OR or Lucy-Rho- OR constructs and lysed in lysis buffer containing 1% NP-40, 150 mM NaCl, 50 mM Tris and 1 mM EDTA on ice for 30 min. The lysate was cleared by centrifugation at 16,000 x g for 30 min at 4^o^C and 10% of the lysate was collected in Laemmli sample buffer for later analysis. Flag-tagged ORs were then immunoprecipitated from the remaining lysate using M2 monoclonal Flag beads. Both the immunopreciptated fraction (B) and unbound fractions (UB) were lysed in Laemmli sample buffer and equal amounts were loaded on a gel along with the input lysate. Proteins were transferred to a nitrocellulose membrane and immunoblotted with the polyclonal Flag antibody using standard procedures. The input lysate membrane was stripped and reprobed for β-actin to ensure equal loading.

### Luciferase Assay

The luciferase assay was performed as previously described [5]. Briefly, ORs were transfected into HEK293T cells along with constructs encoding for CREB-dependent luciferase (Firefly) and a constitutively expressed luciferase (Renilla). OR activation leads to a rise in cAMP which drives an increase in Firefly luciferase expression. Firefly activity is normalized to the activity of the Renilla luciferase to control for variation in cell number and transfection efficiency. Data were collected using a FLUOstar Omega automated platereader (BMG LabTech, Cary, NC). For some experiments, RTP1S was also transfected.

### Statistical Analysis

One-way ANOVA analysis followed by the Student-Newman Keuls test was performed on the luciferase reporter assay to compare the doses of isovaleric acid (0.1-5 mM) or eugenol (100-300 µM) to the non-treated control (0 µM). As each condition was performed in triplicate, the analyses were done with an n=3, and P values ≤ 0.05 were deemed significant. A Student T-Test was performed on the ELISA to assess the significance of the increased protein expression (Control construct vs. Lucy construct for each OR, n=4 for each condition). P values ≤ 0.05 were deemed significant. In both the luciferase reporter assay and the ELISA, the error bars represent the SEM.

## Results

### Surface Expression of ORs with and without Rhodopsin tags

Many ORs remain orphan receptors due to their inability to traffic in *in vitro* assays. To establish the trafficking ability of a varied group of ORs, we cloned 15 diverse ORs, the majority of which are orphan receptors which have not been previously reported to reach the cell surface. Live, non-permeabilized HEK 293T cells were surface labeled with a Flag antibody to detect membrane associated receptor and OR surface expression was scored based on detectable surface immunofluorescence. A ‘+’ was scored for those ORs whose surface expression was detectable in >90% of all fields of view, while ORs received an ‘*’ if surface expression was found in <50% of all fields of view (n=4). A complete lack of detectable surface expression was scored as a ‘-‘(Table 1). Examples of OR surface expression (or absence) can be found in Figure 1, and the results for all ORs are summarized in Table 1 (Columns 1, 3, 5, and 7). ORs were cloned both with and without a 22-amino acid Rho tag (an N-terminal tag often used to aid in surface trafficking of ORs *in vitro* [15]). A small number of ORs were detected on the cell surface even in the absence of the Rho tag (Table 1, column 1). Surprisingly, the addition of the Rho tag (Table 1, column 3) had little effect on the number of ORs which reached the cell surface. Importantly, ‘internal’ Flag staining was consistently seen for every construct (as shown in Figure S1 for Rho-Olfr691 and Rho-hOR52B2).

**Table 1:** Trafficking of olfactory receptors in the absence and presence of RTP1S, Ric8b and G_αolf_ (accessory factors).

**Columns**	**1**	**2**	**3**	**4**	**5**	**6**	**7**	**8**
Flag-tagged Olfactory Receptors	OR		Rho-OR		Lucy-OR		Lucy-Rho-OR	
Chaperones?	None	RTP1S, Ric8b, G_αolf_	None	RTP1S, Ric8b, G_αolf_	None	RTP1S, Ric8b, G_αolf_	None	RTP1S, Ric8b, G_αolf_
Olfr78	+	+	+	+	+	+	+	+
hOR51E2	+	+	+	+	+	+	+	+
mOREG	+	+	+	+	+	+	+	+
Olfr145	*	+	+	+	+	+	+	+
Olfr691	-	+	-	+	+	+	+	+
hOR52B2	-	-	-	-	+	+	+	+
Olfr99	-	-	-	-	+	+	+	+
Olfr693	-	-	-	+	-	-	-	+
Olfr805	-	-	-	-	-	-	*	+
Olfr1392	-	-	-	+	-	+	-	+
Olfr1393	-	-	-	+	-	+	-	+
Olfr90	-	-	-	+	-	+	-	+
Olfr545	-	-	-	-	-	-	-	+
Olfr985	-	-	-	-	-	-	-	+
Olfr394	-	-	-	-	-	-	-	*

- No detectable OR surface expression

+ OR surface expression detected in the majority of fields of view (>90% of all fields of view)

_*_ = OR surface expression detected in a minority of fields of view (<50% of all fields of view)

### A cleavable signal peptide enhances olfactory receptor surface expression

Recently, a 17-amino acid N-terminal signal peptide on Leucine Rich Repeat Containing 32 (LRRC32) was found to be required for proper cell surface expression of LRRC32 in regulatory T cells and HEK293T cells. This sequence (MRPQILLLLALLTLGLA) represents a classic cleavable signal peptide that serves to mediate the integration of proteins into the ER membrane [28]. We hypothesized that the addition of this cleavable peptide, known here as “Lucy” for its leucine rich repeat regions, could also promote surface expression of olfactory receptors; the use of a *cleavable* signal sequence would be advantageous, as it would potentially aid in trafficking without adding additional amino acids to the mature OR protein. To determine whether the Lucy tag can promote OR surface trafficking, we added the Lucy sequence to the N-terminus of the 15 ORs and found that a total of 7 ORs were able to reach the cell surface (Table 1, column 5). To determine if the Lucy and Rho tags may have an additive effect, we assayed for the surface expression of ORs tagged with both Lucy and Rho and found that 8 ORs reached the cell surface (Table 1, column 7).

### Lucy works synergistically with RTP1s and other accessory proteins to promote OR surface expression

Previously, studies have found that the Rho tag works in synergism with RTP1S, Ric8b and G_αolf_ to induce the greatest functional expression of ORs to date [18]. Therefore, we wondered if the Lucy tag could also work synergistically with these accessory proteins. To test for this, OR constructs (both with and without the Rho and Lucy tags) were co-transfected into cells along with RTP1S, Ric8b, G_αolf_ and assayed for detectable surface expression. Examples can be seen in Figure 2 and the results for all ORs are summarized in Table 1 (Columns 2, 4, 6, and 8). For the untagged ORs, co-expression of the accessory proteins allowed for surface expression of 5 ORs (Table 1, column 2). When Rho-tagged ORs were co-expressed with accessory proteins (currently the best practice for achieving OR surface expression [18]), 9 ORs were found on the cell surface (Table 1, column 4). When Lucy-tagged ORs were co-expressed with the accessory proteins, 10 ORs were found on the cell surface (Table 1, column 6). These data confirm that the Rho tag can work synergistically with accessory proteins to promote proper expression [18], and demonstrate that the same is true for the Lucy tag (Table 1). Importantly, when we used Lucy-Rho-tagged ORs together with known chaperones (RTP1S/Ric8b/G_αolf_), all 15 ORs were detected on the cell surface (Table 1, Column 8).

**Figure 3:**
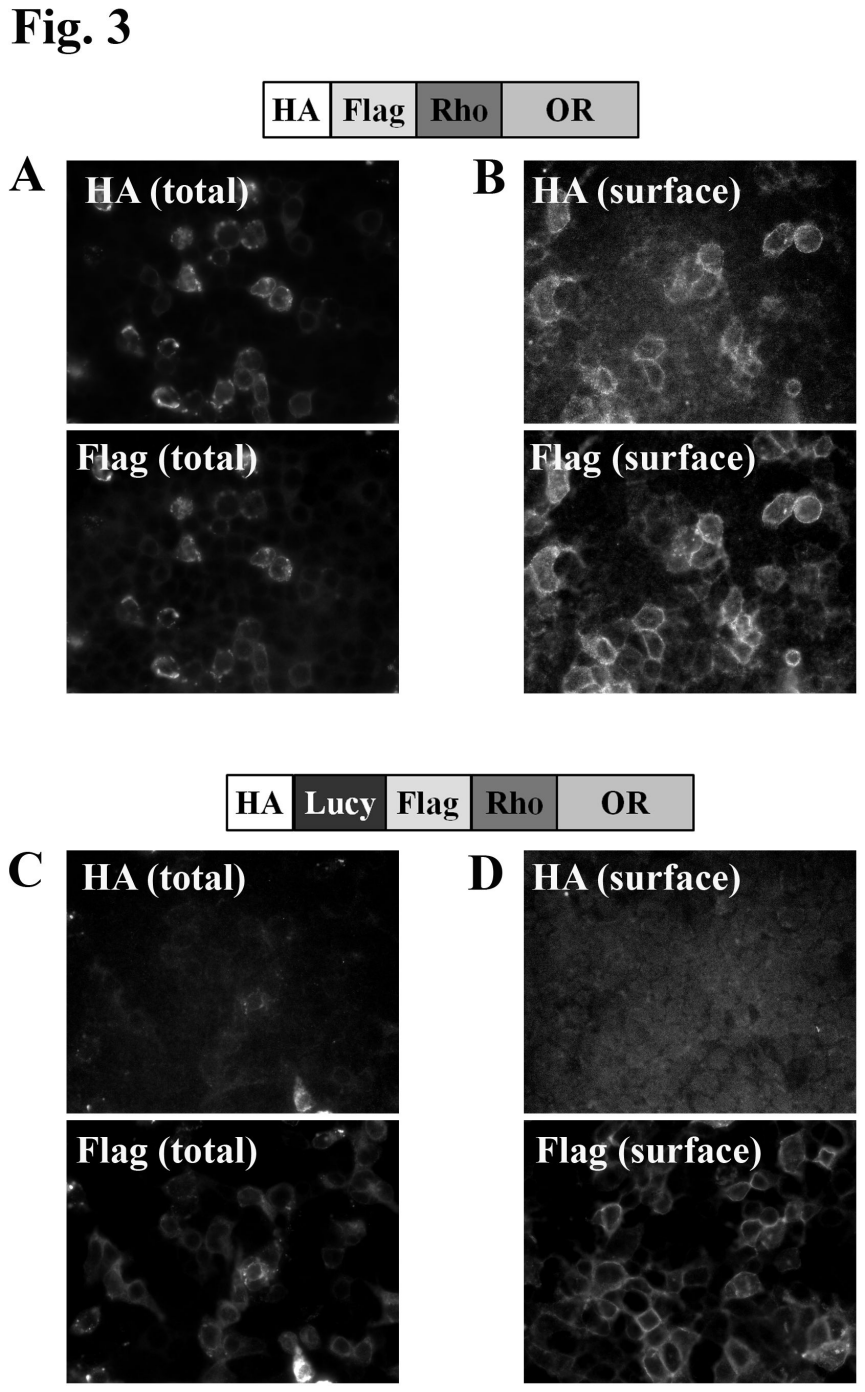
The Lucy tag is a cleavable signal peptide. HA-Flag-Rho-Olfr691 (A and B) or HA-Lucy-Flag-Rho-Olfr691 (C and D) constructs were expressed in HEK293T cells along with RTP1S. Cells were fixed and stained with both an HA and Flag antibody (A and C) to detect total tagged OR or surface labeled with the HA and Flag antibodies (B and D) to detect surface-associated OR. HA surface stain is observed only in the absence of the Lucy tag, indicating a functional Lucy cleavage site.

It is worth noting that although the Rho tag, the Lucy tag, and OR chaperones all promote surface expression of some (but not all) ORs tested, they seem to promote expression of *different* populations of ORs. For example, Olfr99 and hOR52B2 require the Lucy tag for surface expression, but not the combination of accessory proteins or the Rho tag (Table 1). On the other hand, Olfr1393, 1392 and 90 are found on the cell surface only when co-expressed with RTP1S, Ric8b and G_αolf_ and one of the N-terminal tags (Lucy or Rho). Still, other ORs (i.e. Olfr691) properly traffic with either the Lucy tag or the accessory proteins (but not the Rho tag alone). Ideally, one would prefer to achieve surface expression with the minimal amount of modification to the OR protein itself. Importantly, as a classic signal peptide, the Lucy tag contains a putative cleavage site. As such, it is not present on the mature protein which reaches the plasma membrane (as demonstrated below, Figure 3). Using the cleavable Lucy tag (in the absence of the 22-amino acid Rho tag), we are able to achieve surface expression of 10/15 ORs tested (Table 1, Column 6). This will allow for functional characterization of these ORs with only an 8-amino acid flag tag on the plasma membrane protein (previously, only 5 ORs reached the cell surface with a Flag tag alone, Table 1, Column 2).

Finally, although our goal was to achieve surface expression for ORs which did not previously reach the cell surface at all, we noted that some ORs appeared to have enhanced surface expression when both the Lucy tag and the accessory proteins were present (for example, Olfr78 and Olfr691 as seen in Figures 1 and 2). The increase in surface expression was confirmed when the fluorescent surface images were quantitated for Olfr691 (Figure S2).

### The Lucy tag is cleaved

The Lucy tag is a putative cleavable signal peptide found on the N-terminus of LRRC32, and has been previously shown to be cleaved from the mature LRRC32 protein [28]. To determine whether the Lucy tag is also cleaved from the olfactory receptor constructs, we added an HA tag to the extreme N-terminus of both Rho-Olfr691 (Figure 3A,B) and Lucy-Rho-Olfr691 (Figure 3C,D). These constructs were then expressed in HEK293T cells along with RTP1S, which allows for Rho-tagged Olfr691 surface expression. The cells were stained in parallel with both an HA and Flag antibody to detect either the total OR population for both tags (Figure 3A, C), or, on a separate coverslip, the cell surface membrane-associated receptor only for both tags (Figure 3B, D). If the Lucy tag is cleaved, the HA tag should be removed (along with the Lucy tag) early on in the biosynthetic pathway and should not be detectable at the cell surface. When cells expressing HA-Flag-Rho-691 were stained, both Flag and HA antibodies could detect surface-associated (Figure 3A) and intracellular receptor (Figure 3B), indicating that both tags were present on the mature protein. However, when HA-Lucy-Flag-Rho-691 was surface labeled, the HA epitope was no longer present on the cell surface, although surface-associated receptor was still detectable via the Flag tag (Figure 3D). In addition, while there was abundant intracellular Flag staining, there was only weak HA staining (Figure 3C). Taken together, these results indicate that the Lucy tag acts as a functional, cleavable signal peptide when added to the N-terminus of ORs and is likely removed early on in the biosynthetic pathway.

### The Lucy tag increases total protein levels of all ORs

**Figure 4:**
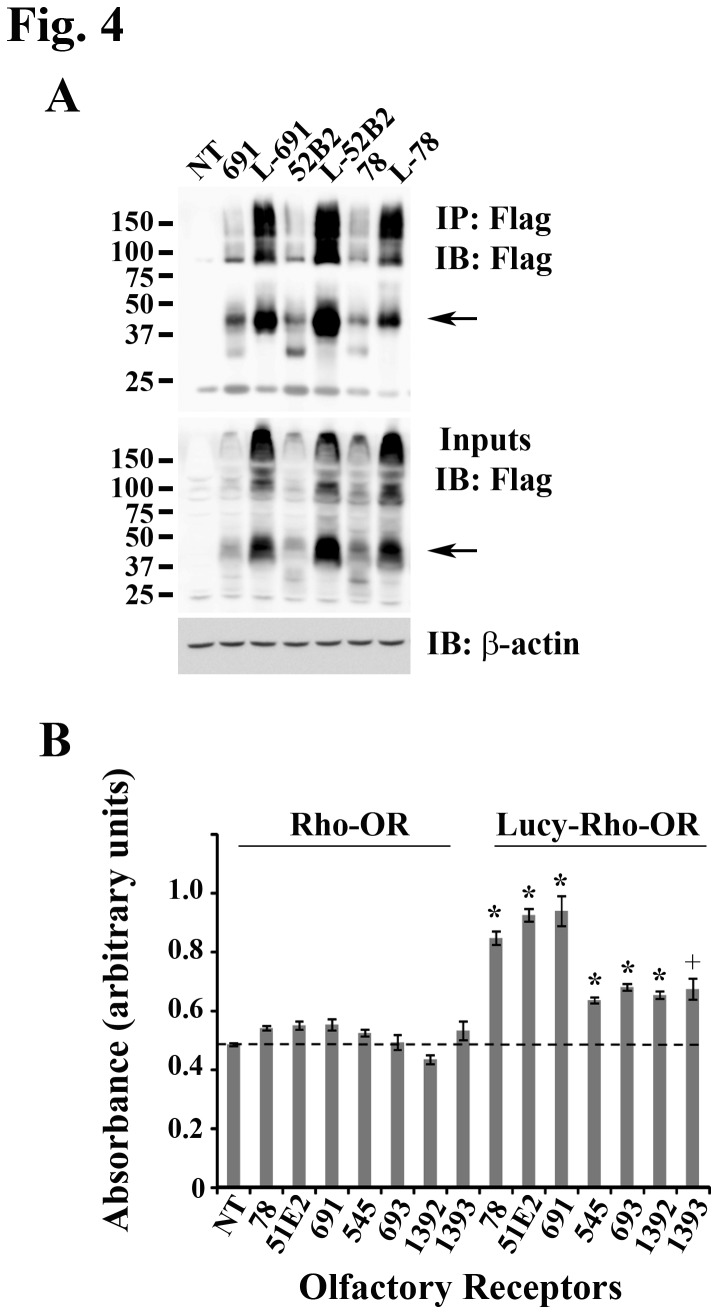
The Lucy tag increases OR protein levels. (A) Rho-tagged and Lucy-Rho-tagged ORs (L-OR) were immunoprecipated from HEK293T cells using monoclonal M2 Flag beads. Bound (B) lysates were immunoblotted with the Flag antibody to detect total OR levels. The arrow indicates the mature OR product at 39 kb. The input was also immunoblotted with the Flag antibody and then stripped and reprobed for β-actin to ensure equal loading. (B) An ELISA was performed for HEK293T cells expressing either Rho-tagged ORs or Lucy-Rho-tagged ORs to detect total OR levels using a monoclonal Flag antibody. Total protein levels are graphed as absorbance in arbitrary units. The dashed line indicates the background as measured by a non-transfected (NT) control. All measurements were performed in quadruplicate and the error bars indicate the SEM. An * represents significance as measured by the student T-test (Rho- OR vs. Lucy-OR) with P ≤ 0.005 and a + represents significance with a P ≤ 0.05. The Lucy tag increased total OR expression of ORs, as shown in both (A) and (B).

When staining HEK293T cells expressing either Rho-ORs or Lucy-Rho-ORs, we noted that there was consistently more surface and intracellular Flag staining with the Lucy tagged constructs (Figure S1). Since Lucy is an ER signal peptide, it is possible that it stabilizes the protein, and thus, increases expression. To examine total protein levels, we transfected HEK293T cells with either Rho- OR or Lucy-Rho- OR constructs, lysed the cells, and immunoprecipitated the OR using M2 Flag beads. An aliquot of the original lysate (input) and the immunoprecipitated ORs were then immunoblotted with a Flag antibody and a subset of ORs are shown in Figure 4A. Typically, ORs are detected as a complex of high-molecular weight bands, likely due to aggregation, degradation and other modifications [8,16], as seen in the whole cell extract (Figure 4A input); immunoprecipitation (Figure 4A IP: Flag) allows for improved resolution. Both the high molecular weight bands and a prominent band at 39 kDa (the predicted size of tagged ORs) were completely recovered in the bound lysate (no bands were detected in the unbound fraction). In both the immunoprecipitate and input lysate, the presence of the Lucy tag appears to increase total OR protein expression (Figure 4A). To ensure equal loading, the immunoblot was stripped and reprobed for β-actin. To quantitate the increase in OR protein, we performed an ELISA to detect total Flag protein levels. As seen in Figure 4B, levels of Rho-ORs were elevated just slightly above background (nontransfected control; dashed line). However, when the Lucy tag was added, protein levels of all ORs tested were increased (1.5-2 fold increase n = 4, P ≤ 0.005), suggesting that the Lucy tag may stabilize the expressed ORs (Figure 4B).

### Lucy does not alter OR-ligand specificity

**Figure 5:**
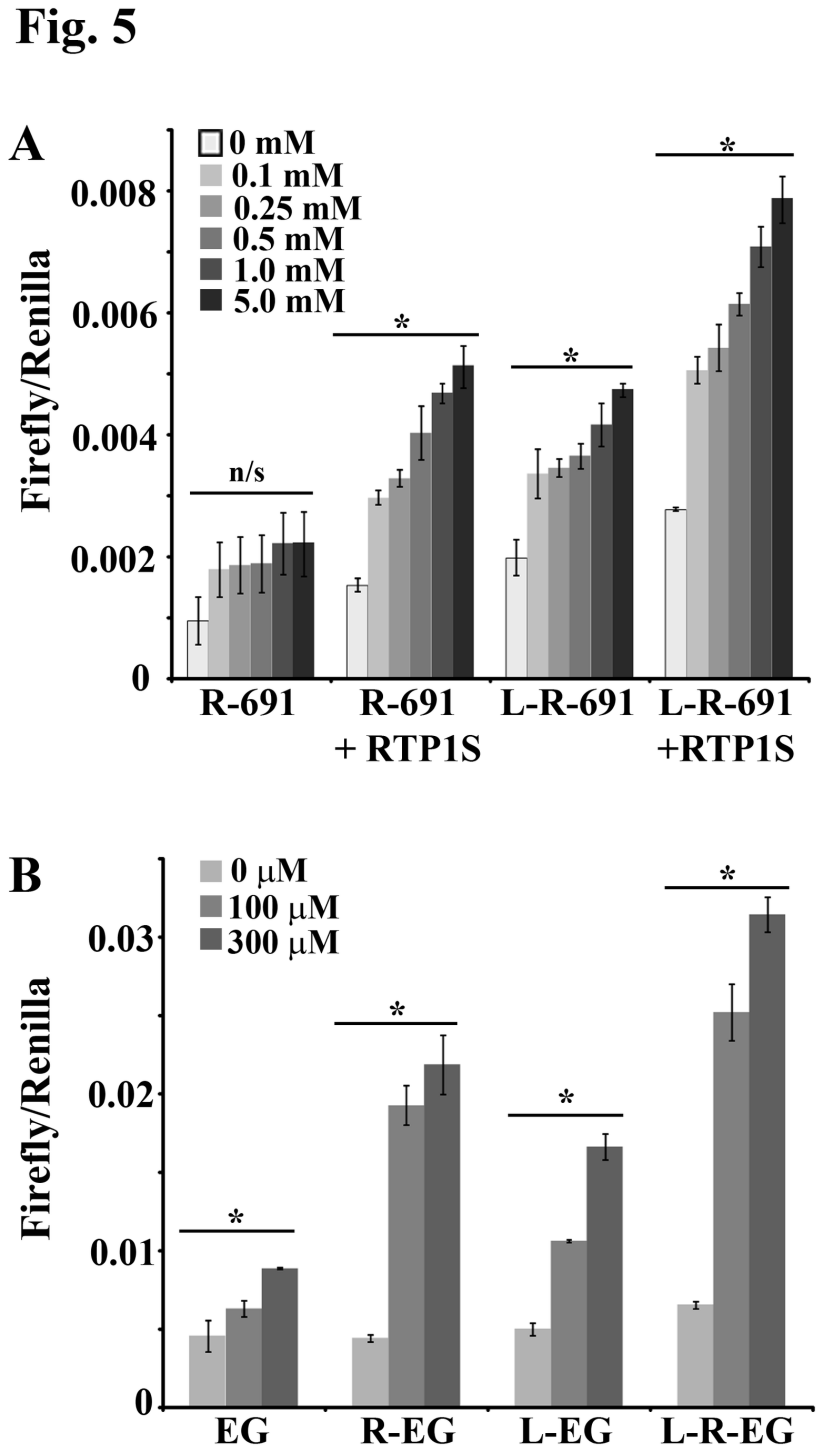
The Lucy tag does not alter OR signaling. (A) A luciferase reporter assay was performed for both Rho-Olfr691(R-691) and Lucy-Rho-Olfr691 (L-R-691) with and without RTP1S. Cells expressing the Olfr691 constructs were grown in a 96-well plate and exposed to the known Olfr691 ligand, isovaleric acid (0-5 mM). Error bars represent the SEM. By ANOVA and Student-Newman-Keuls, no concentrations of isovaleric acid activated R-691 (n/s = no significance). For R-691 + RTP1S, L-691, and L-691 + RTP1S, P ≤ 0.05 for 0 mM vs. all doses of isovaleric acid (marked by an *). In addition, for R-691 + RTP1S, P ≤ 0.05 for 5 vs 0.5, 0.25 and 0.1, 1 vs 0.25 and 0.1, 0.5 vs 0.1. For L-691, P ≤ 0.05 for 5 vs 0.1, 0.25 and 0.5. For L-691 + RTP1S, P ≤ 0.05 for 5 vs 0.5, 0.25 and 0.1, 1 vs 0.5, 0.25 and 0.1, 0.5 vs 0.1. (B) A luciferase reporter assay was performed for mOREG (EG), Rho-mOREG (R-EG), Lucy-mOREG (L-EG) and Lucy-Rho-mOREG (L-R-EG), all in the absence of RTP1S. Cells expressing the mOREG constructs were grown in a 96-well plate and exposed to the known mOREG ligand, eugenol (100-300 µM). Error bars represent the SEM. By ANOVA and Student-Newman-Keuls, all concentrations of eugenol significantly activated (P ≤ 0.05) eg, R-EG, L-EG and L-R-EG as compared to 0 µM (marked by an *). In addition, 100 and 300 µM eugenol were significant from each other (P ≤ 0.05) for both L-EG and L-R-EG. In both A and B, the Firefly: Renilla ratio was measured and compared to the non-treated control. An increase in the ratio indicates OR activation. Both Lucy-Rho-691 and Lucy-tagged mOREG constructs were activated with their ligands indicating that the Lucy tag does not alter OR signaling.

OR surface expression in heterologous cell systems is a prerequisite for further functional studies, including OR deorphanization. Since Lucy is a cleaved signal peptide, it is not incorporated into the mature protein and thus, should not interfere with OR-ligand specificity or downstream signaling. However, some data have suggested that ORs require a “co-receptor” (RTP1S) to signal properly [19]. To determine whether Lucy-tagged ORs expressed on the cell surface can still respond to their ligands in the absence of RTP1S, and to ensure that the Lucy tag is not altering ligand binding or detection, we assayed for a functional ligand response using a luciferase reporter assay [5]. In this assay, OR-ligand binding leads to an increase in cAMP which drives the expression of a CRE luciferase. An increase in the Firefly (CRE-dependent luciferase): Renilla (constitutively activated luciferase) ratio indicates OR activation. Previously, it was determined that Olfr691 responds to isovaleric acid [16]. Here, Rho-Olfr691 and Lucy-Rho-Olfr691 were expressed in HEK293T cells with or without RTP1S and exposed to 0.1-5 mM isovaleric acid. By itself, Rho-Olfr691 was not significantly activated at any concentration (Figure 5A), confirming the lack of detectable surface expression as seen by immunofluorescence (Figure 1). When Rho-Olfr691 was co-expressed with RTP1S, we observed a dose-dependent increase in the Firefly: Renilla ratio (P ≤ 0.002 for all doses compared to 0 mM), confirming the previous findings (Figure 5A). Lucy-Rho-Olfr691 also responded to isovaleric acid in a dose-dependent manner with and without the addition of RTP1S (P ≤ 0.007 for all doses compared to 0 mM) confirming that the surface expression observed in Figures 1 and 2 represents functional protein. As seen in Figure 5A, we found that OR constructs with higher surface expression (whether due to the Lucy tag, Rho tag, or chaperones) tended to have a higher Firefly/Renilla ratio at baseline (non-treated, NT) in the luciferase reporter assay. This often corresponded to a higher ratio with stimulation as well, implying that the increased baseline may indicate a low level of basal signaling in the absence of ligand.

To confirm that the Lucy tag does not interfere with OR-ligand binding and downstream signaling and that RTP1S is not required for proper activation, we also performed the luciferase reporter assay on mOREG (Figure 5B). mOREG is a well-characterized OR known to respond to eugenol (hence its name). As mOREG is one of the ORs that reach the cell surface under every condition tested, we expressed the OR with and without the Lucy and Rho tags and assessed its response to eugenol. Every permeation of the mOREG construct resulted in a dose-dependent activation with eugenol (P ≤ 0.05, 300 µM vs 0 µM), once again suggesting that the cleavable Lucy tag does not interfere with OR-ligand binding and that properly trafficked ORs likely do not require a co-receptor for function.

## Discussion

The limiting factor in OR deorphanization has been the ability – or, often, the inability – to heterologously express ORs on the cell surface. Here, we report that the addition of a leucine rich cleavable signal peptide (Lucy tag) onto the N-terminus of ORs significantly improves detectable surface expression, as well as total protein expression. When combined with RTP1S, Ric8b and G_αolf_, we found that all 15 of the Lucy-ORs that we examined successfully trafficked to the surface, providing promise for future deorphanization and other functional studies.

### What is the mechanism of the Lucy tag?

Cleavable signal peptides are natively found on secreted proteins and subsets of TMD proteins (including some GPCRs) [25,26]. Recognition of these cleavable peptides at the extreme N-terminus of the protein by the Signal Recognition Particle (SRP) promotes co-translational ER translocation and ensures that the complete mature protein is translated in the lumen of the ER (as opposed to the cytosol) [25,26]. When TMD proteins do not contain a cleavable signal peptide, one of the TMDs (usually the first) takes its place and acts as a signal anchor sequence. Therefore, a signal peptide at the N-terminus of proteins is not required for proper ER translocation and only 5-10% of GPCRs possess a classic signal peptide [26]. Typically, receptors that utilize a signal peptide have long N-terminal tails that can rapidly fold, preventing post-translational translocation across the ER membrane [26]. ORs do not have unusually long N-terminal tails, and it is possible that the co-translational ER entry via the Lucy tag helps to stabilize the receptor or prevents misfolding. In support of this, our preliminary studies showed that mRNA levels of Lucy and non-Lucy-tagged constructs were similar, despite the fact that total protein expression for all ORs was enhanced by the addition of the Lucy tag (Figure 4). However, in addition to increasing total protein levels, the Lucy tag by itself promoted surface expression of some ORs (Figure 1). This indicates that the Lucy tag has roles beyond protein translation and may be promoting some of the later steps of protein trafficking or ER exit. ORs do not natively possess signal peptides, but they are not retained in the ER when natively expressed in the olfactory epithelium (OE); thus, the Lucy tag must be helping ORs to overcome ER processing problems that are unique to heterologous expression. Although the Lucy tag does increase total expression, this increase does not appear to account for the increase in surface expression. In preliminary studies, we found that simply transfecting more of a Rho-tagged OR (in µg), did not correlate with increased surface expression. Therefore, the increase in both total and surface expression for Lucy-tagged ORs is unique to the tag itself.

It should be noted that the Lucy tag may be dependent on the immediate upstream sequence of the mature protein. Mutational studies have shown that signal peptides and their adjacent N-terminal sequences act as a “functional unit” and deletion of this upstream domain negatively affects ER translocation [26,35]. As our Lucy-tagged constructs contained a Flag tag (for detection purposes) following the cleavable peptide, the presence of this tag may be required for proper function and cleavage. Preliminary studies found that deletion of the Flag tag from a Lucy-Flag-OR construct prevented the OR from responding to its ligand, suggesting impaired surface expression (without the Flag tag, surface expression could not be assayed independently in this construct). It appears, then, that like other N-terminal sequences [26,35], the ‘context’ of the Lucy tag may be important for its function.

### Multiple Blockage Steps for OR Trafficking

Studies have shown that ORs are retained in the ER when expressed in heterologous cells where they undergo ER-associated degradation [8,13,36,37]. However, as demonstrated by Wu, et al [19], the ER-Golgi transition step is not the only point of retention. This is evidenced in our work by the fact that, under the same conditions, some ORs are more efficiently trafficked to the cell surface than others, despite taking what is presumed to be a common route to the plasma membrane. For example, in this study Olfr78, hOR51E2 and mOREG trafficked to the cell surface even in the absence of the Rho tag. While the surface expression was relatively weak, mOREG does respond its ligand in the absence of the Rho tag (Figure 5B). Others, however, required much more assistance to make it to the plasma membrane. Olfr545, Olfr985 and Olfr394 required both the Lucy and Rho tags as well as the co-transfection of RTP1S, Ric8b and G_αolf_ for proper surface expression. What can account for these differences? It is likely that some ORs are retained at multiple cellular checkpoints and each of these tags and accessory proteins function at one (or more than one) of these points. Further studies are clearly required to fully understand OR trafficking and the Lucy tag could prove to be a valuable tool to answer these questions.

### What is the function of RTP1S?

The current “gold standard” in OR trafficking was found to be the combination of a Rho tag with RTP1S, Ric8b and G_αolf_ [18]. Of these three accessory proteins, RTP1S is thought to be the most crucial as it can promote OR surface expression even in the absence of the other two compounds for some ORs [5,15,16,22–24]. Indeed, we have found that RTP1S is often required for cell surface expression. By contrast, G_αolf_ is typically not required, and Ric8b is only occasionally necessary. RTP1S is natively expressed in the olfactory epithelium, and thus, it has been speculated that it is necessary for OR expression in both heterologous cells as well as the OE [16,18]. Recently, Wu et al performed a series of mutations and substitutions to RTP1S in order to elucidate the mechanism(s) of this important protein [19]. From this study, it was concluded that RTP1S and olfactory receptors (in this case Olfr599) interact throughout the biosynthetic pathway, and that individual domains of RTP1S are required for different stages of OR trafficking. It was also speculated that RTP1S may function as a co-receptor as the localization of both the OR and RTP1S to lipid raft domains was required for OR activation [19]. Indeed, much of our data is consistent with this study. Many of the ORs that we examined required RTP1S (with Ric8b and G_αolf_) for proper surface expression (i.e. Olfr693, Olfr1392, Olfr1393, Olfr90 and Olfr545). However, we also found that Olfr78, hOR51E2 and mOREG (Fig 5B) were able to respond to their ligand even in the absence of RTP1S. To ensure that RTP is not natively expressed in HEK 293T cells, we performed RT-PCR using primers that could detect both the long and short form of RTP but did not detect any band in HEK293T cells (a positive control performed simultaneously gave a band of the expected size; Fig. S3). In addition, Olfr691 was functionally expressed on the cell surface (with the Lucy tag) in the absence of RTP1S, and retained a normal ligand response (Fig. 5A). While RTP1S is clearly playing important roles in the early trafficking steps and folding of olfactory receptors, it is not required for OR activation or surface expression and therefore is not an obligate co-receptor. Use of the Lucy tag can help shed new light on the functions of RTP1S, as OR function can now be assayed both with and without RTP1S (Figure 5).

### Potential for deorphanization

OR deorphanization has been greatly hampered by the inability to functionally express ORs in heterologous cell systems. To date, the addition of N-terminal tags or the co-expression of chaperone proteins has been crucial for surface expression of many receptors, but has not allowed for widespread OR deorphanization. In this study, we examined the trafficking of 15 diverse ORs with the Lucy tag, many of which are orphan receptors. Because the Lucy tag is cleaved prior to surface expression, this tag does not interfere with or alter ligand binding. In fact, for many ORs, the addition of the cleavable Lucy tag allowed surface expression even in the absence of the 22-amino acid Rho tag (Figures 1 and 2), allowing for the potential of OR deorphanization with only an 8 amino acid Flag tag on the mature protein. The identification of ligands for ORs is becoming increasingly important and has implications beyond olfaction. It has recently been demonstrated that ORs are expressed in multiple tissues outside of the OE [31,38–43], where they play functional roles in processes as varied as muscle cell migration, renal function, and sperm chemotaxis. In order to understand the roles that ORs are playing both in the OE and in other tissues, ligand assignment is imperative. The addition of the Lucy tag represents a distinct improvement in the trafficking of heterologously expressed ORs which we hope will lead to future wide scale deorphanization studies.

## Supporting Information

Figure S1 The Lucy tag promotes detectable surface expression of olfactory receptors in the absence of accessory proteins. (A and B) HEK293T cells were transfected for 24 h with Rho-tagged or Lucy-Rho-tagged Olfr691 (A) or its human homologue, hOR52B2 (B). Cells were surface labeled with a polyclonal Flag antibody to detect surface-associated OR (a and b) and then fixed, permeabilized and stained with a monoclonal Flag antibody to detect the internal OR population (c and d). Both Olfr691 and hOR52B2 traffic to the surface with but not without the Lucy tag.
(TIF)

Figure S2 The Lucy tag and accessory proteins increase the amount of Olfr691 detected on the cell surface. Surface-labeled Olfr691 was quantitated by measuring the mean fluorescence intensity for each image. This graph represents the mean fluorescence intensity normalized to the corresponding binary nuclear image for the same field of view (surface/nuclear). Error bars represent the SEM, and ‘+ chaperones’ indicates the presence of RTP1S, Ric8b and G_αolf_. Representative images corresponding to each condition are pictured below the graph showing the increased surface expression. For all conditions that promoted surface expression (Flag-Rho-691 + chaperones, Lucy-Flag-Rho-691 and Lucy-Flag-Rho-691 + chaperones), there was a significant increase in the surface/nuclear ratio as compared to Flag-Rho-691 (*P ≤ 0.01 as measured by ANOVA and Student-Newman Keuls). In addition, the fluorescence for Lucy-Flag-Rho-691 + chaperones was significantly increased as compared to both Lucy-Flag-Rho-691 and Flag-Rho-691 + chaperones (P ≤ 0.001).
(TIF)

Figure S3 HEK293T cells do not natively express RTP. HEK293T and whole kidney RNA was reverse transcribed with (+) or without (-) reverse transcriptase and PCR was performed using primers for both the long and short form of RTP. Amplified RTP had an expected size of 548 bp. RTP was amplified from kidney cDNA but not from HEK293T cDNA.
(TIF)
