# Supplementary figures and images for: A Cleavable N-Terminal Signal Peptide Promotes Widespread Olfactory Receptor Surface Expression in HEK293T Cells
Source: PLoS One. 2013 Jul 1;8(7):e68758. doi: 10.1371/journal.pone.0068758 (PMC3698168; doi:10.1371/journal.pone.0068758)

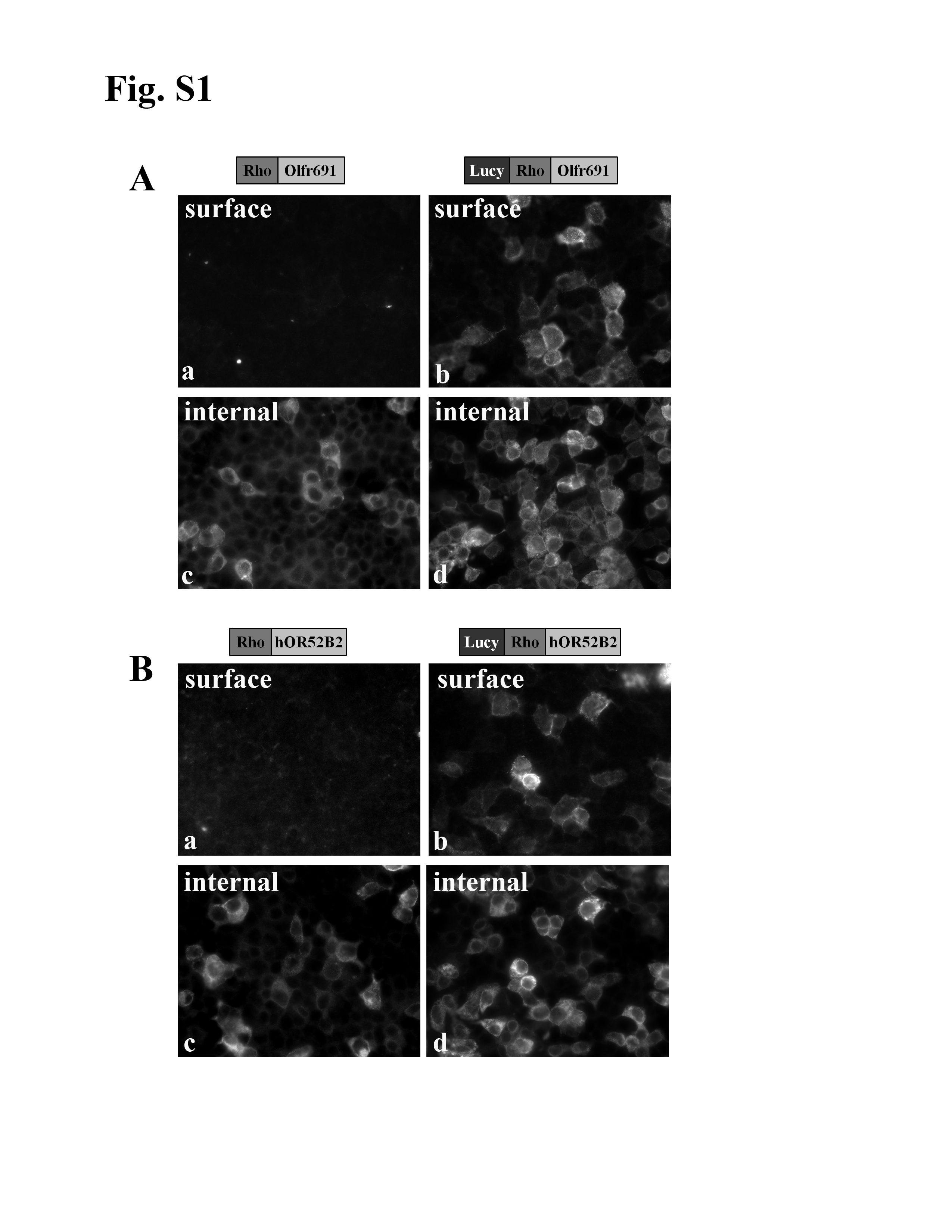

Supplement: Figure S1 — (A and B) HEK293T cells were transfected for 24 h with Rho-tagged or Lucy-Rho-tagged Olfr691 (A) or its human homologue, hOR52B2 (B). Cells were surface labeled with a polyclonal Flag antibody to detect surface-associated OR (a and b) and then fixed, permeabilized and stained with a monoclonal Flag antibody to detect the internal OR population (c and d). Both Olfr691 and hOR52B2 traffic to the surface with but not without the Lucy tag. (TIF) [file pone.0068758.s001.tif]

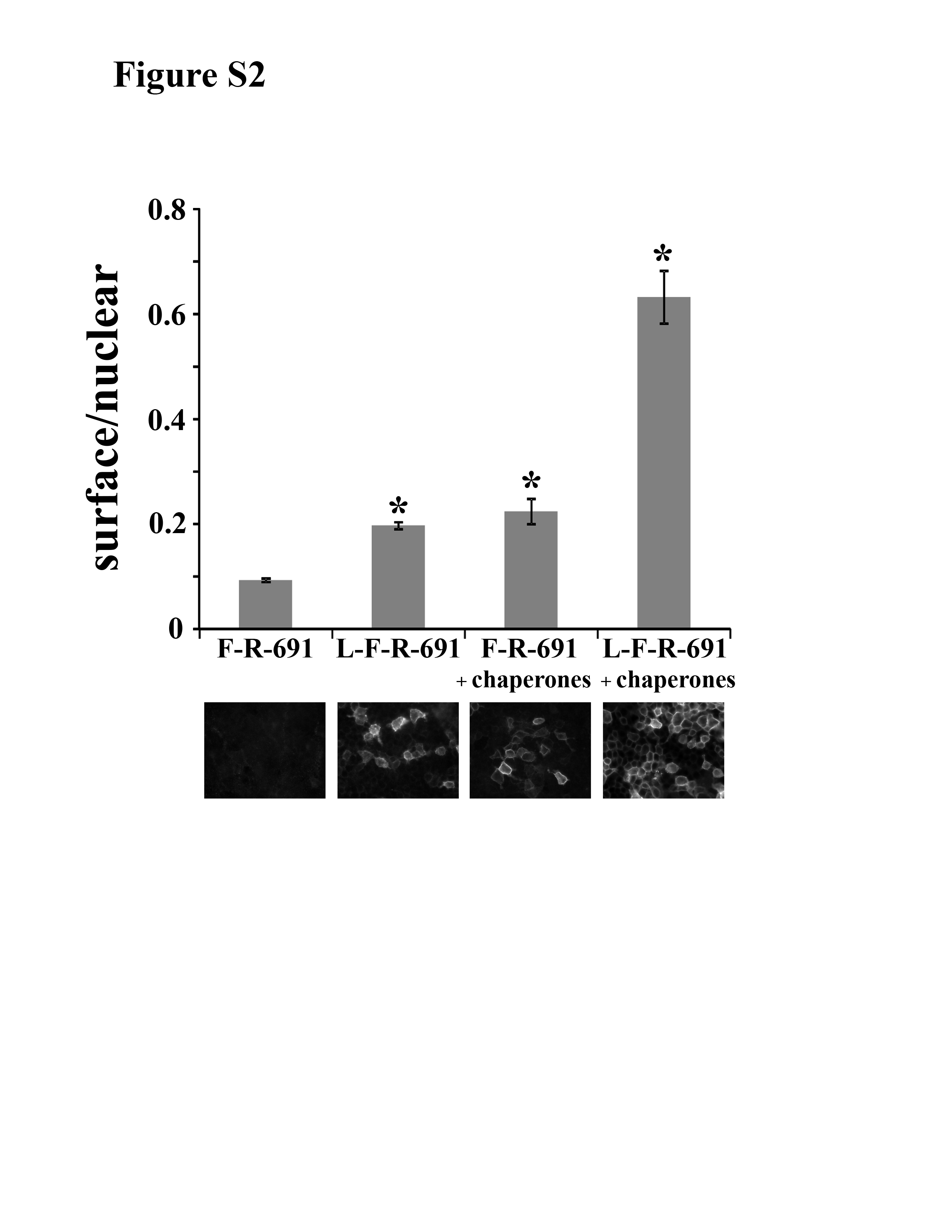

Supplement: Figure S2 — Surface-labeled Olfr691 was quantitated by measuring the mean fluorescence intensity for each image. This graph represents the mean fluorescence intensity normalized to the corresponding binary nuclear image for the same field of view (surface/nuclear). Error bars represent the SEM, and ‘+ chaperones’ indicates the presence of RTP1S, Ric8b and Gαolf. Representative images corresponding to each condition are pictured below the graph showing the increased surface expression. For all conditions that promoted surface expression (Flag-Rho-691 + chaperones, Lucy-Flag-Rho-691 and Lucy-Flag-Rho-691 + chaperones), there was a significant increase in the surface/nuclear ratio as compared to Flag-Rho-691 (*P ≤ 0.01 as measured by ANOVA and Student-Newman Keuls). In addition, the fluorescence for Lucy-Flag-Rho-691 + chaperones was significantly increased as compared to both Lucy-Flag-Rho-691 and Flag-Rho-691 + chaperones (P ≤ 0.001). (TIF) [file pone.0068758.s002.tif]

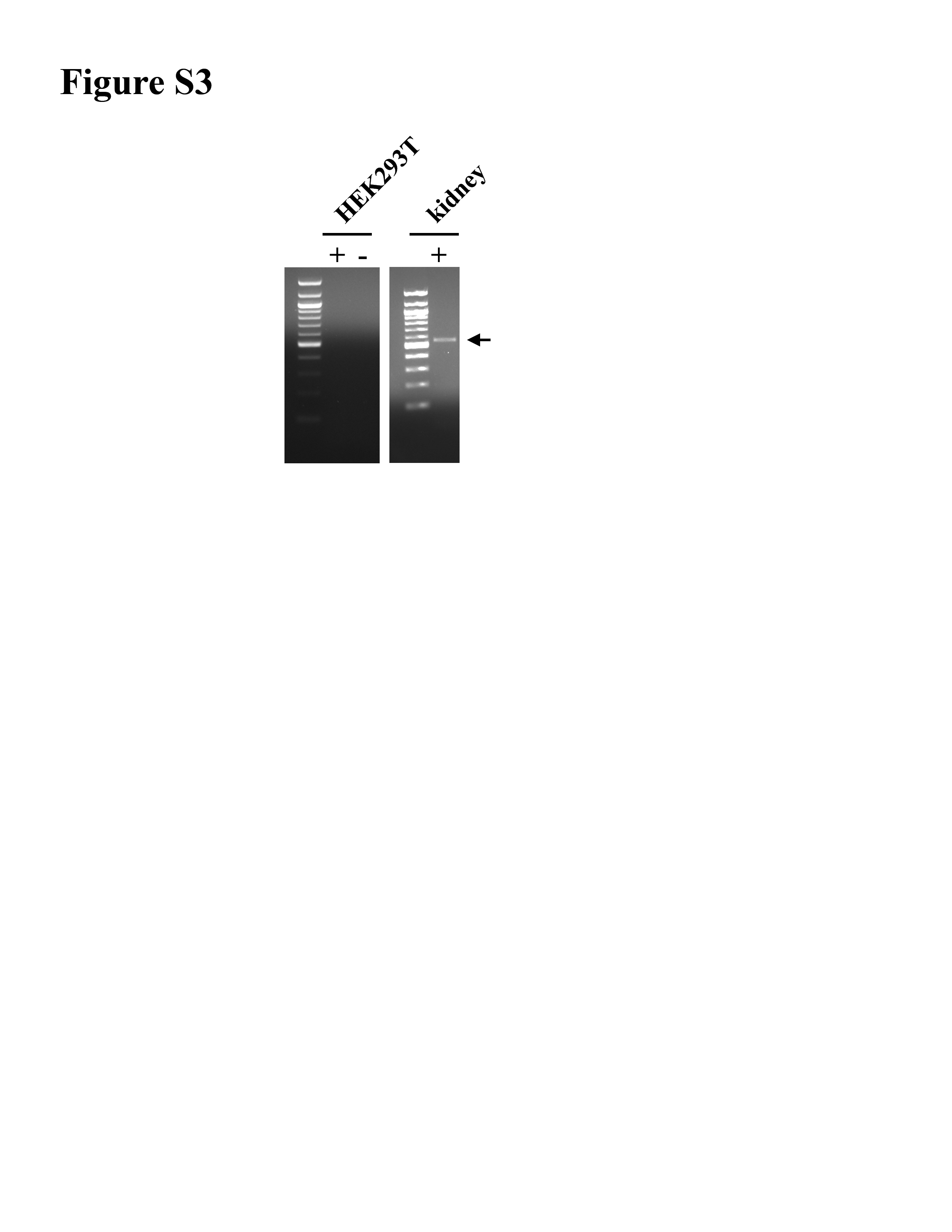

Supplement: Figure S3 — HEK293T and whole kidney RNA was reverse transcribed with (+) or without (-) reverse transcriptase and PCR was performed using primers for both the long and short form of RTP. Amplified RTP had an expected size of 548 bp. RTP was amplified from kidney cDNA but not from HEK293T cDNA. (TIF) [file pone.0068758.s003.tif]
